# Pharmacodynamics and Pharmacokinetics of Ublituximab Compared with Other Anti-CD20 Monoclonal Antibodies for Multiple Sclerosis Treatment

**DOI:** 10.2174/011570159X392955250815095236

**Published:** 2025-08-26

**Authors:** Monica Margoni, Luca Battistini, Diego Centonze, Roberto Furlan, Claudio Gasperini, Massimo Filippi

**Affiliations:** 1 Neuroimaging Research Unit, Division of Neuroscience, IRCCS San Raffaele Scientific Institute, Milan, Italy;; 2 Neurology Unit, IRCCS San Raffaele Scientific Institute, Milan, Italy;; 3 Neurorehabilitation Unit, IRCCS San Raffaele Scientific Institute, Milan, Italy;; 4 Neuroimmunology Unit, IRCCS Fondazione Santa Lucia, Rome, 00179, Italy;; 5 Department of Systems Medicine, Tor Vergata University, Rome, Italy;; 6 Unit of Neurology, IRCCS Neuromed, Pozzilli (IS), Italy;; 7 Vita-Salute San Raffaele University, Milan, Italy;; 8 Institute of Experimental Neurology, Division of Neuroscience, IRCCS Ospedale San Raffaele, Milan, Italy;; 9 Neurological Division, S. Camillo Forlanini Hospital, Rome, Italy;; 10 Neurophysiology Service, IRCCS San Raffaele Scientific Institute, Milan, Italy

**Keywords:** Multiple sclerosis, anti-CD20 therapy, ublituximab, ocrelizumab, ofatumumab, disease-modifying therapy, B cells

## Abstract

The therapeutic scenario for multiple sclerosis (MS) has expanded rapidly over the last few years. Among the available treatments, anti-CD20 monoclonal antibodies, including rituximab, ocrelizumab, ofatumumab, and ublituximab, have shown significant results in reducing disease activity and slowing progression, particularly in relapsing MS. The distinct mechanisms of action, including the pharmacokinetic and pharmacodynamic profiles as well as the immunogenicity of these drugs, require careful consideration to tailor treatment for individual patients. A comprehensive review of the literature was conducted by searching PubMed and evaluating key studies, trials, and congress abstracts related to the use of anti-CD20 monoclonal antibodies. The analysis focused on the pharmacokinetic and pharmacodynamic profiles, as well as the immunogenicity, of anti-CD20 therapies currently available, with particular emphasis on the recently approved ublituximab. Ocrelizumab is effective in both relapsing-remitting and primary-progressive MS, using antibody-dependent cellular cytotoxicity (ADCC) as its primary mechanism of action, with intravenous and subcutaneous administration options ensuring flexible treatment delivery. Ofatumumab depletes B-cells through enhanced complement-dependent cytotoxicity, offering convenient monthly subcutaneous self-administration. Ublituximab’s unique glycoengineered fragment crystallizable region enhances ADCC, resulting in rapid B-cell depletion and potentially improving its safety profile. Ublituximab allows for a shorter infusion time without requiring post-infusion monitoring after the second dose, provided there have been no prior reactions. Understanding the characteristics of different anti-CD20 monoclonal antibodies is critical for optimizing treatment, enhancing patient outcomes, and minimizing treatment burden. Ublituximab represents a promising option, offering a shorter infusion time and higher ADCC activity, which complements existing treatments such as ocrelizumab and ofatumumab.

## 1 INTRODUCTION

Multiple sclerosis (MS) is a chronic inflammatory, demyelinating, and neurodegenerative disorder of the central nervous system (CNS), often leading to progressive disability [1]. The MS treatment has rapidly expanded, with over twenty disease-modifying therapies (DMTs) now at disposal. Among these, anti-CD20 monoclonal antibodies, which mediate B-cell depletion, stand out for their strong efficacy and favorable safety profile [2-5]. Approved agents include rituximab (off-label), ocrelizumab, ofatumumab, and ublituximab, with ocrelizumab being the only option for primary progressive (PP) MS. The recent approval of ublituximab highlights the need to understand the differences in pharmacokinetics, pharmacodynamics, and immunogenicity among these anti-CD20 antibodies to support individualized treatment decisions and optimize patient outcomes.

## 2 METHODS

This article summarizes the latest findings on the role of B cells in the immune-pathophysiology of MS, as well as the main findings investigating the pharmacokinetic and pharmacodynamic profiles and immunogenicity of anti-CD20 therapies currently available.

Sources for this review were selected by conducting searches on PubMed using specific keywords ‘antibody dependent cellular cytotoxicity’, ‘anti-drug antibodies’, ‘antigen-presenting cell’, ‘area under the curve’, ‘B cell’, ‘CD19’, ‘CD20’, ‘cerebrospinal fluid’, ‘depleting therapy(ies)’, ‘disease-modifying’, ‘epitopes’, ‘FcγRIIIa’, ‘immunoglobulin’, ‘immunology’, ‘immunogenicity’, ‘lymphocyte’, ‘mechanism of action’, ‘monoclonal antibody(ies)’, ‘multiple sclerosis’, ‘neutralizing antibodies’, ‘ocrelizumab’, ‘ofatumumab’, ‘peak concentration’, ‘pharmacokinetics’, ‘pharmacodynamics’, ‘pathology’, ‘phenotype(s)’, ‘primary progressive’, ‘relapsing-remitting’, ‘rituximab’, ‘secondary progressive’, ‘slowly-expanding lesions’, ‘T cell’, ‘terminal half-life’, ‘trogocytosis’, ‘ublituximab’, from 15 November 1990 to 15 November 2024. Additional articles were selected through searches in the authors' archives. Abstracts from major conferences in the field were also assessed. Only studies published in English were considered. The final reference list was compiled based on originality and relevance to the overall scope of this article.

## 3 B-CELLS IN MS IMMUNE-PATHOPHYSIOLOGY

MS was once thought to be primarily driven by T-cell-mediated mechanisms. Still, growing evidence highlights the significant role of B cells and their interaction with T cells in disease pathogenesis [6, 7]. Different studies have demonstrated the co-localization of B- and T-cell infiltrates with active lesions, as well as the presence of plasma cells and CD20+ B cells in the perivenular spaces of patients with progressive MS [8, 9]. Subpial demyelination, neuronal loss, and cortical thinning are associated with B-cell aggregates in the meninges, some of which resemble follicle-like structures [10, 11].

Recent findings suggest B-cell maturation and immune responses occur both peripherally and within the CNS [12]. B-cell development begins in the bone marrow with antigen-independent maturation, followed by antigen-dependent maturation in peripheral lymphoid tissues [13]. Pre-B cells (CD19^+^, CD20^+^) mature into immature B cells expressing IgM. Upon encountering an antigen and receiving co-stimulation, they undergo further maturation. Isotype switching typically occurs in germinal centers, with the resulting cells migrating to tissues such as the bone marrow, brain, gut, and spleen. B cells differentiate into memory B cells or plasmablasts, guided by chemokines such as CXCL12, CCL25, and CCL28. Beyond antibody production, B cells contribute to antigen presentation and cytokine secretion, amplifying inflammation [14]. Within germinal centers, B cells interact with T helper (Th) cells, which support the formation of memory B cells and the activation of Th cells. In MS, peripheral B cells may escape regulation by dysfunctional T regulatory cells, promoting autoimmunity [12]. Pathogenic B and T cells cross the blood-brain barrier *via* chemokine receptors and adhesion molecules, become reactivated in the CNS, and drive inflammatory and neurodegenerative processes. Though precise mechanisms remain unclear, both cell types play central roles in immune dysregulation in MS.

## 4 ROLE OF ANTI-CD20 ANTIBODIES AND EXPRESSION

### 4.1 Focus on CD20: Expression and Function in B Cells

CD20 is a transmembrane phosphoprotein that is not glycosylated and has a molecular weight between 33 and 37 kDa. It is mainly found on the cell surface throughout most stages of the B-cell lifecycle, except in stem cells, pro-B cells, and fully differentiated plasmablasts and plasma cells [15].

Although its function has still to be fully recognized, CD20 is hypothesized to function either as an ion channel or indirectly regulate calcium release through the B-cell antigen receptor [16, 17]. A recent study demonstrated that CD20 serves as an essential gatekeeper of a membrane nanodomain and the resting state of naive B cells [18]. The loss of CD20 on human B cells resulted in a dissolution of the IgD-class nanocluster (composed of CD19 and CD20) and a transient B cell activation, inducing a B cell-to-plasmacell differentiation [18].

### 4.2 Expression (Trogocytosis) and Function in a Small Population of T Cells and Potential Relevance in MS

Recent studies have highlighted a possible role of anti-CD20 monoclonal antibodies in modulating T-cell responses [19]. A small subset of T cells acquires CD20 from B cells through trogocytosis during activation by an antigen-presenting B cell, making them potential targets for anti-CD20 therapies [19]. B-cell depletion not only removes CD20+ T cells directly but also alters T-cell populations by disrupting B-T cell interactions [20-24]. One study showed CD3^+^CD20^+^ T cells dropped from 2.4 ± 0.36% to 0.04 ± 0.01% (mean ± standard error of the mean [SEM]) of CD45^+^ lymphocytes after two weeks of ocrelizumab treatment [23]; similar depletion was observed with rituximab over 12 weeks [22]. Ublituximab treatment increased the number of naive CD4^+^ and CD8^+^ T cells, while reducing the number of effector and central memory T cells. Th1 cells declined and regulatory T cells increased, suggesting improved immune regulation [24]. Ofatumumab also reduced peripheral CD20^+^ T cells and enhanced T cell regulation, lowering autoreactivity and migration of T cells [20]. Ofatumumab decreased non-suppressive regulatory cells and increased naive regulatory T cells while adjusting follicular helper/regulatory T cell ratios in MS patients compared to controls [21]. Similar effects on T cells were reported with ocrelizumab [23]. While the clinical relevance of CD20^+^ T cell depletion remains under investigation, these findings suggest that it may contribute to the therapeutic efficacy of anti-CD20 therapies [22-24].

## 5 COMPARATIVE PHARMACOLOGY OF UBLITUXIMAB COMPARED WITH OTHER ANTI-CD 20 THERAPIES

### 5.1 Anti-CD20 Drugs Overview

Among the different anti-CD20 monoclonal antibodies, only three are officially approved for the treatment of MS: ocrelizumab, ofatumumab, and, more recently, ublituximab. Ocrelizumab is a humanized monoclonal antibody that is approved for both relapsing-remitting MS (RRMS) and PPMS based on the results of the phase III trials OPERA I/II and ORATORIO [3, 5, 25, 26]. Ocrelizumab was approved for intravenous administration at a dose of 300 mg over more than 2.5 hours for the first two doses, with subsequent doses of 600 mg delivered over more than 3.5 hours every 6 months. Pre-medication is recommended before each infusion to mitigate systemic reactions. A shorter infusion time (*i.e*., 2.5 hours) was approved for the 600 mg doses [27]. Most recently, the subcutaneous administration of ocrelizumab at a dose of 920 mg every 6 months has been approved for both relapsing MS (RMS) (*i.e*., RRMS, and active secondary progressive MS- SPMS) and PPMS treatment, following the II/III OCARINA trial results [28].

Ofatumumab is a fully human monoclonal antibody approved for treating relapsing forms of MS, including clinically isolated syndrome (CIS), RRMS, and active SPMS, following the completion of the phase III ASCLEPIOS I and II trials [4]. It is self-administered *via* subcutaneous injection of 20 mg once monthly (three initial doses administered weekly starting at week 0, followed by once-monthly dosing starting at week 4). It does not require premedication, providing a more convenient self-administration option for patients.

Ublituximab is a murine-human glycoengineered chimeric monoclonal antibody recently approved for the treatment of RMS in the completed phase II/III trials ULTIMATE I and II [29, 30]. Ublituximab was assessed in phase III trials with an initial 150 mg infusion over 4 hours, followed by a 450 mg infusion over 1 hour 2 weeks later, and subsequently administered every 24 weeks until week 96 [29]. Premedication was necessary before each infusion in both phase II and III studies [30]. It was administered *via* different routes (orally, intravenously, intramuscularly, or subcutaneously) to reduce the frequency and severity of infusion-related reactions. After the second administration, according to the summary of product characteristics, no post-infusion monitoring is required for patients who did not experience infusion-related reactions at the first infusion.

Although rituximab, a chimeric monoclonal antibody, is not specifically approved for MS by regulatory agencies, it is used off-label for MS treatment.

These anti-CD20 therapies have revolutionized the treatment landscape for MS by providing highly effective options for disease management.

### 5.2 Structural Features and Epitope Binding of Anti-CD20 Monoclonal Antibodies

**Fig. (1):**
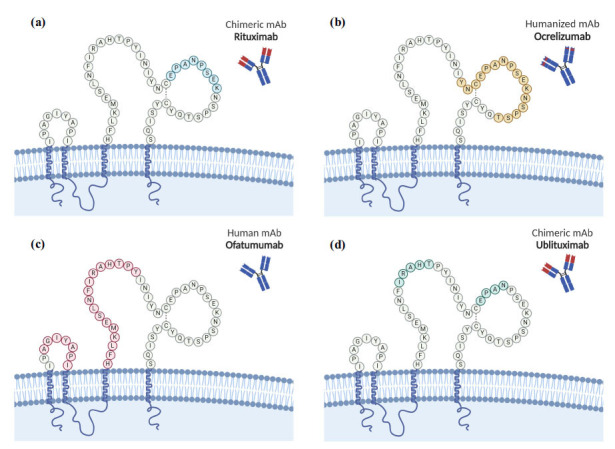
Anti-CD20 monoclonal antibodies target epitopes. (**a**) Rituximab, a chimeric antibody, binds to amino acid residues 165-182 on the large extracellular loop of CD20. (**b**) Ocrelizumab, a humanized antibody, targets residues 165-180 on the same loop. (**c**) Ofatumumab, a fully human antibody, interacts with sequences in both the small (residues 74-80) and large extracellular loops of CD20. (**d**) Ublituximab binds to residues 155-159 and 168-171 on the large extracellular loop of CD20. **Abbreviation**. mAb=monoclonal antibody.

The distinct molecular structures and binding epitopes of anti-CD20 monoclonal antibodies play a significant role in differentiating their primary mechanisms of action (Figs. **1a**-**d**, Table **1**).

The structure of these monoclonal antibodies can be divided into different key components. The variable region, the fragment antigen-binding (Fab) region, is responsible for the specific binding to the CD20 antigen. The mediation of immune responses occurs through an interplay involving the fragment crystallizable (Fc) region of the antibody on the surface of immune effector cells [31, 32]. The binding of the Fc region to these receptors mediates mechanisms such as antibody-dependent cellular cytotoxicity (ADCC), complement-dependent cytotoxicity (CDC), and antibody-dependent cellular phagocytosis (ADCP). Glycosylation of the Fc region is a critical structural feature that affects the antibody’s stability, solubility, and interaction with Fc receptors. Variations in glycosylation can significantly influence the binding affinity of the antibody to Fcγ receptors on immune cells, thereby modulating its effector functions.

Rituximab and ocrelizumab both recognize highly similar and overlapping CD20 epitopes around amino acid residues 165-180 on the large extracellular loop (Fig. **1a**, Table **1**) [32, 33]. Besides this similarity, ocrelizumab primarily depletes B cells through ADCC, with a minor contribution from CDC, whereas rituximab predominantly determines higher levels of CDC compared to ADCC [34]. These variations in the primary mechanisms of action likely stem from differences in the Fc regions of the two antibodies, with ocrelizumab exhibiting enhanced binding to low-affinity variants of FcγRIIIa [34, 35]. Ocrelizumab is subject to Fc receptor amino acid engineering to enhance ADCC. Ofatumumab binds to a unique epitope distinct from those of rituximab and ocrelizumab, partially overlapping with the epitope targeted by ublituximab (Fig. **1c**, Table **1**). Besides targeting a region of the large extracellular loop located N-terminally to the rituximab/ocrelizumab epitope, it also binds to a seven-residue region within the small extracellular loop [32, 33]. This epitope is believed to contribute to ofatumumab’s stronger and more stable binding to CD20 as well as its slower off-rate compared to rituximab and ocrelizumab [36, 37]. These factors are thought to improve CDC by promoting stronger binding and more effective accumulation of complement system components on the surface of the target cell [32]. Ofatumumab’s mediation of CDC is less dependent on cell-surface density—a key factor typically required for effective CDC—compared to rituximab. Ofatumumab has demonstrated higher CDC activity than rituximab, ocrelizumab, and ublituximab [38-40]. Ofatumumab has been found to determine ADCC at levels twice as high as rituximab, comparable to ocrelizumab but lower than ublituximab [33, 41]. The epitope targeted by ublituximab spans two distinct regions of the large extracellular loop, with a mechanism of action that predominantly relies on ADCC rather than CDC [30, 32, 33]. Studies have shown that ublituximab elicits higher ADCC activity than rituximab, ocrelizumab, and ofatumumab [33]. This enhanced activity is largely attributed to its glycoengineered Fc region that has been afucosylated to improve FcγRIIIa binding and optimize the recruitment of immune effector cells [42]. Monoclonal antibodies primarily relying on ADCC, such as ublituximab and ocrelizumab, are particularly influenced by Fcγ receptor polymorphisms. The main genotypic variation of the FcγRIIIa receptor is the single nucleotide substitution from G to T at cDNA nucleotide position 559 of the FCGR3A gene generating two different FcγRIIIa allotypes: one with a valine (V) and one with a phenylalanine (F) at amino acid position 158, known as FcγRIIIA-V158F polymorphism (rs396991) [43-45]. A recent study revealed that the most common FcγRIIIa-158 polymorphism at nucleotide position 5093 was the G/T genotype (55% of patients), followed by the T/T genotype in 38% and the G/G genotype in 7% of the analyzed patients [46]. The T/T genotype results in an F/F phenotype, T/G in a V/F phenotype, whereas G/G results in a V/V phenotype. The presence of a valine (V/V or V/F) has been shown to enhance the natural killer (NK) cell’s binding affinity to an IgG1 or IgG3 antibody as compared to the presence of a homozygous phenylalanine genotype (F/F), resulting in a higher level of NK cell-mediated ADCC [44, 45, 47]. These polymorphisms should be clinically considered as their presence can significantly impact the effectiveness of treatment [48-51]. Previous studies performed in both patients with neuromyelitis optica spectrum disorder and rheumatoid arthritis showed that the presence of the F/F genotype was associated with a higher relapse risk while receiving rituximab treatment [52-54]. While ocrelizumab and ublituximab have a high affinity for this polymorphism [12], rituximab has a lower affinity, thus not recruiting this mechanism with 158F (the low-affinity variant of the Fc receptor) [55]. Compared to ocrelizumab, rituximab, and ofatumumab, ublituximab demonstrates a 16- to 25-fold higher binding affinity for FcγRIIIa-158V and a 10- to 22-fold higher binding affinity for FcγRIIIa-158F, respectively [56].

### 5.3 B-cell Depletion and Replenishment

Clinical trials have demonstrated that anti-CD20 monoclonal antibodies lead to substantial improvements in both clinical and imaging outcomes, outperforming both placebo and active treatment alternatives [2-4, 26, 29, 30, 57-60].

According to previous studies [2, 61], in the phase II/III OLYMPUS trial, a rapid and nearly complete (>95%) depletion of CD19+ B cells was detected two weeks after the initial 1000 mg rituximab infusion, with this low level sustained until the study’s conclusion at week 96 [57]. In the Phase III ORATORIO trial, intravenous ocrelizumab, administered every 24 weeks, led to nearly complete depletion of CD19+ B-cell populations within two weeks of the initial 600 mg dose, with this depletion persisting throughout the 216-week study period [3]. The Phase III OPERA trials assessed an equivalent dosing regimen of ocrelizumab, and the results were comparable [26]. The ASCLEPIOS, APLIOS, and APOLITOS trials evaluated the efficacy of subcutaneous ofatumumab at the identical dosing protocol and initial loading doses, leading to an effective and sustained depletion of B-cells within 1-2 weeks [4, 58, 62, 63]. In the phase III ASCLEPIOS trials, more than 92% of patients maintained B-cell levels below the lower limit of normal (LLN) at every visit from week 2 until the study’s completion at week 120 [4]. In the phase II APLIOS trial, all patients exhibited B-cell levels below the LLN (80 cells/μL) within two weeks after initiating atumumab treatment [62]. In the phase II APOLITOS study, B-cell depletion below the LLN was observed as early as day 7 and persisted through week 24 [58]. In a phase II trial evaluating intravenous ublituximab, CD19+ B-cell depletion was observed in most patients within 24 hours of the first 150 mg infusion, with mean B-cell levels decreasing significantly from 7.3% at baseline to 0.2% within 24 hours post-infusion [30]. No significant B-cell repletion was recorded, and this reduction was maintained throughout the 48-week study [30]. In the phase III ULTIMATE trials, a 96% reduction in the median number of CD19+ B cells was observed within 24 hours of the initial 150 mg dose of ublituximab [29]. The faster depletion observed with ublituximab may have a positive impact on inflammatory activity, allowing a timelier therapeutic switch.

It is unclear whether complete B-cell depletion is essential for achieving therapeutic efficacy in people with MS or whether similar clinical outcomes could be achieved with partial B-cell depletion, thus enabling the use of lower dosing regimens [33]. While treatment ensures sustained and thorough B-cell depletion, B-cell recovery has been found to occur more rapidly after discontinuing subcutaneous ofatumumab compared to other anti-CD20 therapies, including its intravenous formulation [33, 60, 64]. B-cell levels have been observed to rise above the lower LLN (40 cells/μL) or baseline in ≥50% of patients within 24-36 weeks (median 24.6 weeks) after discontinuation of subcutaneous ofatumumab [64]. Pharmacokinetic studies of B-cell repletion support this observation, estimating a median recovery time of 23 weeks [65]. A direct comparison revealed median B-cell repletion times to the lower LLN or baseline for other anti-CD20 monoclonal antibodies, such as ocrelizumab (72 weeks, range 27-175 weeks) and rituximab (12-16 months) in patients with rheumatoid arthritis [66]. Ublituximab showed a similar median time for CD19+ B-cell counts to return to baseline or the lower LLN (100 cells/μL), with a median of 70.3 weeks (range 0.1-75.1 weeks). In comparison, subcutaneous ofatumumab demonstrated faster repletion rates than intravenous high-dose ofatumumab (100, 300, or 700 mg), with only a few patients showing recovery above the LLN (100 cells/mm^3^) after 48 weeks [60]. In the intravenous ofatumumab study, B-cell repopulation was observed in all patients within 104 weeks after treatment was stopped [60]. The OLYMPUS trial revealed that 26 weeks following the discontinuation of rituximab therapy, only 35% of patients treated with rituximab had reached the threshold [60].

The faster B-cell repletion seen with subcutaneous ofatumumab could provide clinical benefits in specific scenarios, offering greater flexibility in managing infections, administering vaccinations, or promptly resuming therapy following a temporary break [64]. B-cell repletion between doses has not been observed with ofatumumab, being attributed to its more frequent administration of lower doses compared to other anti-CD20 therapies [65]. Partial B-cell repletion has been noted between the semi-annual infusions of ocrelizumab, although the clinical significance of this remains unclear [67]. No association was found between elevated B-cell levels and the so-called ocrelizumab “wearing-off” phenomenon [68].

Unlike MS, in other conditions, dosages of anti-CD20 monoclonal antibodies are frequently adjusted based on a patient's body weight. Fixed-dose administration can result in variations in pharmacokinetics and pharmacodynamics across a population, leading to differences in therapeutic exposure and efficacy due to variations in body weight [69]. In MS, a pharmacokinetic study investigating ocrelizumab predicted that a body-weight-adjusted dose would result in lower therapeutic exposure compared to a fixed-dose regimen. It is crucial to assess whether B-cell depletion and repletion kinetics are significantly influenced by patient body weight [70]. In patients with a lower body mass index (BMI) of <25 kg/m^2^, who had higher exposure to ocrelizumab, the treatment benefit in reducing confirmed disability progression at 12 and 24 weeks was greater compared to those with a higher BMI (>25 kg/m^2^). When considering new or enlarged T2 lesions as an endpoint, the treatment benefit was more pronounced in patients with a higher BMI compared to those with a lower BMI. A study analyzing subgroup efficacy data from the OPERA I and II trials found that increased ocrelizumab exposure may reduce MS disability progression [71]. A study showed that B-cell depletion with ofatumumab was largely independent of body weight effects on pharmacokinetics and depletion was consistently maintained in patients regardless of their weight [65]. The study did report an association between decreasing body weight and longer B-cell repletion times to the lower LLN (40 cells/μL), with repletion times of 128 days for a weight of 110 kg, 164 days for 70 kg, and 204 days for 50 kg [65]. In line with these findings, an analysis of the ASCLEPIOS I and II trials found no clear association between body weight variability and differences in treatment efficacy, particularly regarding progression independent of relapse activity [72]. No clinically significant differences in ublituximab pharmacokinetics were reported with respect to age, sex, body weight, or mild hepatic or renal impairment [73].

A more thorough analysis could have important implications for dosing strategies in certain anti-CD20 monoclonal antibody therapies. These findings underscore the importance of evaluating multiple efficacy endpoints before drawing definitive conclusions about the relationship between body weight and treatment efficacy.

### 5.4 Pharmacokinetics and its Relevance to Pharmacodynamic Profiles of Anti-CD20 Monoclonal Antibodies

The pharmacokinetics of anti-CD20 antibodies are crucial for optimizing dosing schedules and achieving therapeutic efficacy while minimizing adverse effects in people with MS.

The intravenous administration of rituximab, ocrelizumab, and ublituximab ensures 100% bioavailability, as the drugs enter circulation directly. Subcutaneous ofatumumab, on the other hand, has a slow absorption rate with peak concentrations (C_max_) occurring approximately 3-7 days post-injection, depending on the dose. After administration, anti-CD20 antibodies distribute primarily within the vascular and interstitial compartments, targeting CD20-expressing B cells. The volumes of distribution for ocrelizumab and ublituximab are approximately 2.78 L and 2.18 L, respectively, which are relatively low compared to small molecules due to the large size of monoclonal antibodies, limiting their penetration into tissues [73, 74]. Ofatumumab has a larger volume of distribution (5.42 L) due to its subcutaneous administration, resulting in slower and more prolonged absorption [75].

For ocrelizumab, the C_max_ is reached by the end of the infusion period, which is typically 4 to 6 hours [74]. At the standard dose of 600 mg, the ocrelizumab C_max_ value ranges between 200 and 300 µg/mL, depending on patient variability. For ofatumumab, the C_max_ is significantly lower, typically ranging from 1.43 to 3.45 µg/mL, reflecting the slower absorption through the subcutaneous route [75]. In phase II studies investigating ublituximab, the Cmax was 140 µg/mL, and the median C_max_ ratio of week 24 to day 1 was 3.04, in keeping with a three-fold increase in dose and indicative of no accumulation of the drug. Similarly, the C_max_ ratio of week 48 to week 24 was 1, also suggesting a lack of significant accumulation [73].

The area under the curve (AUC) is a key parameter representing the total exposure of the drug over time. For ocrelizumab, the AUC after a 600 mg dose in people with MS ranges from 15,000 to 20,000 µg·h/mL, depending on patient factors such as body weight and disease status. Ofatumumab has an AUC range of approximately 480-940 µg·h/mL after a single dose. For ublituximab, the mean AUC is 3000 μg/mL/d at steady state. The AUC is indicative of the amount of drug available to interact with its target over time. It is crucial for maintaining adequate B-cell depletion in MS. Ocrelizumab has a clearance rate of approximately 0.17 L/day, and its terminal half-life (t½) is around 26 days [74]. Rituximab and ublituximab showed t½ of 22 days [73]. Conversely, the t½ of ofatumumab is 16 days (Table **1**) [75]. Both target-mediated drug disposition and the overall B-cell load influence the t½ of anti-CD20 monoclonal antibodies. As B-cell depletion occurs, the clearance rate slows, resulting in a prolonged half-life of the drug in circulation. Ofatumumab, administered subcutaneously, has a similar clearance rate; however, due to its different administration route, the half-life is slightly shorter, approximately 16 days [75].

The different pharmacokinetic profiles of the anti-CD20 monoclonal antibodies may significantly influence their clinical dynamic profiles. Monoclonal antibodies such as rituximab, ocrelizumab, and ublituximab, administered intravenously, reach high peak and mean blood concentration levels. These drugs may effectively target B-cell populations in different anatomical compartments, including meningeal follicles, where B-cell-driven immune processes contribute to MS pathology [10, 11]. Supporting this hypothesis, an experimental study demonstrated that the intrathecal administration of anti-CD20 antibodies effectively reduced the density of CNS meningeal B cells [76], suggesting a potential benefit of intravenous therapies in controlling disease progression by reducing B-cell activity within the CNS. Interestingly, a recent study showed that higher exposure to ocrelizumab was associated with a reduced rate of disability progression [77]. In patients with RMS, an incomplete B-cell depletion or higher B-cell counts in blood before the next infusion showed a trend toward elevated risk of disease progression [77]. This suggests that maintaining lower B-cell levels over a longer time may lead to better disability outcomes.

### 5.5 Administration Route

Treatment burden is becoming an increasingly significant factor in clinical decision-making [78]. The various administration routes for anti-CD20 monoclonal antibodies may impact the quality of life for MS patients. The approved regimens for rituximab, ocrelizumab, and ublituximab involve intravenous infusions along with premedication and/or post-dose monitoring, taking several hours. Ublituximab stands out among anti-CD20 monoclonal antibody therapies as it can be administered within 1 hour. In the pooled analysis of the ULTIMATE studies, 96.6% of participants completed ublituximab infusions without interruption [79]. Premedication can be administered through various routes, including oral, intravenous, intramuscular, or subcutaneous. Post-infusion monitoring is not required after the second administration for patients who did not experience infusion-related reactions during the initial infusion, thereby reducing hospitalization time.

Ofatumumab is approved for RMS as a self-administered subcutaneous injection, using either a pre-filled syringe or the autoinjector pen, following an initial dose under healthcare supervision (Table **1**). A phase III clinical trial demonstrated the noninferiority of subcutaneous administration compared to intravenous administration of ocrelizumab for the primary endpoint, based on pharmacokinetic measures of serum ocrelizumab levels over 12 weeks, in both RMS and PPMS patients [28]. A similar efficacy was observed for subcutaneous ocrelizumab compared to intravenous ocrelizumab [28]. Although there is growing interest among patients and clinicians in shifting toward subcutaneous administration for monoclonal antibody therapies, due to potential benefits such as enhanced patient adherence and reduced financial strain on healthcare systems [78], some patients may benefit from intravenous infusion in a hospital setting for different reasons. The prospect of not having the subcutaneous drug stored in their fridge every month can be a significant relief as it minimizes the constant reminder of their illness in their daily environment. Avoiding the need to travel with the medication can reduce logistical burdens and potential stress. This approach also allows patients to focus on their condition less frequently, potentially only twice a year. This can enhance their overall quality of life and mental well-being by reducing the psychological weight of managing their disease.

Different treatment regimens result in varying levels of clinical visits, treatment burden, and convenience, all of which affect flexibility and patient independence. Treatment selection should take into account these health-related quality-of-life factors in addition to considerations of efficacy, safety, and tolerability.

### 5.6 Immunogenicity

The development of anti-drug antibodies (ADAs), particularly neutralizing antibodies (NAbs), poses a significant challenge in the clinical management of patients receiving anti-CD20 monoclonal antibodies. These immunogenic responses can attenuate therapeutic efficacy by blocking the biological interaction with its target, accelerating drug clearance, and diminishing pharmacodynamic effects. Neutralizing antibodies (Nabs) are particularly concerning as they directly inhibit the drug’s mechanism of action, potentially leading to treatment failure or disease exacerbation. ADAs may provoke hypersensitivity reactions, increase the risk of infusion-related adverse events, and compromise long-term safety profiles. These concerns underscore the importance of early detection and monitoring of immunogenicity to optimize therapeutic outcomes. In the phase II/III OLYMPUS trial for PPMS and the phase II HERMES trial for RRMS, 7.0% (20 out of 286) and 24.1% (14 out of 58) of rituximab-treated patients, respectively, tested positive for anti-chimeric antibodies [2, 57]. Relatively high levels of ADAs were also observed in a phase I rituximab trial for RRMS, where 28.6% (6 out of 21) of patients tested positive (Table **1**) [61]. In the ORATORIO phase III trial for PPMS, 1.9% (9 out of 486) of patients developed ADAs and 0.2% (1 out of 486) developed NAbs [3]. Similarly, in the phase III OPERA I and II trials for RMS, 0.4% (3 out of 825) of patients developed ADAs while 0.1% (1 out of 825) developed Nabs [26]. In the phase III ASCLEPIOS I and II studies for RMS, ADAs were observed in 0.2% (2 out of 946) of patients, with no cases of NAbs reported [4].

In the phase III ULTIMATE trials, 17.8% of ublituximab-treated patients tested positive for ADAs at baseline, while 86.5% tested positive at least once during the study. The presence of ADAs had no observable impact on the safety or efficacy of ublituximab. For NAbs, 2.4% tested positive at baseline, with 6.4% testing positive at any point thereafter (Table **1**) [73]. The development of treatment-emergent ADAs and NAbs peaked at week 24 and subsequently declined.

The biological and clinical impact of ADAs on the efficacy and safety of treatments remains unclear, as no definitive evidence has established that the development of these antibodies consistently affects treatment outcomes or safety profiles negatively [2, 57]. For ublituximab, the presence of ADAs or NAbs had no observable impact on B-cell depletion, drug safety, or efficacy. Furthermore, no associations were found between ADA or NAb development and participants' baseline characteristics, including age, sex, race, BMI, and other factors [80]. Studies on rituximab have indicated that ADAs do not have any significant impact on its efficacy or safety profile [81]. Previous reports have suggested a potential involvement of ADAs in serum sickness after rituximab or ocrelizumab treatment [82, 83].

ADA positivity across trials can be related to various factors, including sample preparation, assay features, drug interference, other medications, and MS phenotype. Consequently, comparisons between trials may not be reliable [33].

## CONCLUSION

Over the past decade, anti-CD20 monoclonal antibodies have significantly reshaped the therapeutic landscape of MS, demonstrating strong efficacy in reducing inflammatory activity, slowing disability progression, and improving long-term patient outcomes [5, 25]. Therapies such as rituximab, ocrelizumab, ofatumumab, and ublituximab each offer distinct pharmacologic profiles and administration routes, allowing clinicians to tailor treatment strategies to the individual needs of patients. The recent approval of ublituximab, with its shorter infusion time [56] and enhanced ADCC activity [56], adds another effective tool to the therapeutic arsenal against MS. By enhancing ADCC, ublituximab more effectively recruits immune effector cells, such as NK cells, resulting in rapid and efficient B-cell depletion. This mechanism may also contribute to an improved safety profile, improving patient tolerability and providing an important therapeutic option. Its comparison with rituximab, ocrelizumab, and ofatumumab underscores the differences in mechanisms of action, infusion times, and B-cell depletion profiles, all of which are important factors in individualizing treatment. The distinct administration routes, dosing regimens, and pharmacokinetics/pharmacodynamics profiles of these anti-CD20 therapies are critical to optimizing patient care and minimizing treatment burden. As the therapeutic armamentarium for MS continues to evolve, understanding the nuanced differences between these therapies is essential for maximizing clinical outcomes and ensuring tailored treatments that meet individual patient needs. Future directions should include real-world evidence on long-term safety, head-to-head studies, and the evaluation of biomarkers predictive of treatment response. These efforts will help refine patient selection, personalize therapy, and further improve the overall management of MS.

## Figures and Tables

**Table 1:** Comparison of molecular structure, CD20 epitope, mechanisms of action, route of administration and pharmacokinetic features of the different anti-CD20 monoclonal antibodies for multiple sclerosis.

-	**Rituximab**	**Ocrelizumab**	**Ofatumumab**	**Ublituximab**
Structure	Chimeric murine/ human IgG1	Recombinant humanized glycosylated IgG1	Fully human IgG1	Chimeric IgG1 with glycoengineered Fc segment
CD20 epitope	Amino acid residues 168-175 on large extracellular loop	Amino acid residues 165-180 on large extracellular loop	Sequences of the small (residues 74-80) and large extracellular loops (residues 145-161)	Amino acid residues 168-171 and 155-159 on large extracellular loop
Mechanisms of action	CDC>ADCC	ADCC>CDC	CDC>ADCC	ADCC>CDC
Route of administration	Intravenous	Intravenous	Subcutaneous	Intravenous
Terminal half-life	22 days	26 days	16 days	22 days
Immunogenicity	Most	Less	Least	Less

**Abbreviations**. ADCC=antibody-dependent cellular cytotoxicity; CDC=complement-dependent cytotoxicity; IgG=immunoglobulin.

## LIST OF ABBREVIATIONS

ADAs: Anti-drug Antibodies
ADCC: Antibody-dependent Cellular Cytotoxicity
ADCP: Antibody-dependent Cellular Phagocytosis
AUC: Area Under the Curve
BMI: Body Mass Index
CDC: Complement-dependent Cytotoxicity
CIS: Clinically Isolated Syndrome
CNS: Central Nervous System
DMTs: Disease-modifying Therapies
Fab: Fragment Antigen-binding
Fc: Fragment Crystallizable
LLN: Lower Limit of Normal
MS: Multiple Sclerosis
Nabs: Neutralizing Antibodies
PPMS: Primary Progressive MS
RMS: Relapsing MS
RRMS: Relapsing-remitting MS
SEM: Standard Error of the Mean
SPMS: Secondary Progressive MS
